# Comprehensive Primer Design for Analysis of Population Genetics in Non-Sequenced Organisms

**DOI:** 10.1371/journal.pone.0032314

**Published:** 2012-02-24

**Authors:** Ayumi Tezuka, Noe Matsushima, Yoriko Nemoto, Hiroshi D. Akashi, Masakado Kawata, Takashi Makino

**Affiliations:** Department of Ecology and Evolutionary Biology, Graduate School of Science, Tohoku University, Sendai, Japan; The Centre for Research and Technology, Hellas, Greece

## Abstract

Nuclear sequence markers are useful tool for the study of the history of populations and adaptation. However, it is not easy to obtain multiple nuclear primers for organisms with poor or no genomic sequence information. Here we used the genomes of organisms that have been fully sequenced to design comprehensive sets of primers to amplify polymorphic genomic fragments of multiple nuclear genes in non-sequenced organisms. First, we identified a large number of candidate polymorphic regions that were flanked on each side by conserved regions in the reference genomes. We then designed primers based on these conserved sequences and examined whether the primers could be used to amplify sequences in target species, montane brown frog (*Rana ornativentris*), anole lizard (*Anolis sagrei*), guppy (*Poecilia reticulata*), and fruit fly (*Drosophila melanogaster*), for population genetic analysis. We successfully obtained polymorphic markers for all target species studied. In addition, we found that sequence identities of the regions between the primer sites in the reference genomes affected the experimental success of DNA amplification and identification of polymorphic loci in the target genomes, and that exonic primers had a higher success rate than intronic primers in amplifying readable sequences. We conclude that this comparative genomic approach is a time- and cost-effective way to obtain polymorphic markers for non-sequenced organisms, and that it will contribute to the further development of evolutionary ecology and population genetics for non-sequenced organisms, aiding in the understanding of the genetic basis of adaptation.

## Introduction

Understanding evolutionary processes through genomics approaches is essential for the study of a number of important issues, including the effects of climate change [1]–[5], invasion of alien species [6], and endangerment of species by emerging diseases [7]–[9]. Not only fully-sequenced species but also non-sequenced species are important for these research issues. In molecular ecological studies of non-sequenced organisms, neutral molecular markers such as mitochondrial DNA, microsatellites, and amplified restriction fragment polymorphisms have been routinely used. Although these markers are effective for estimating genetic population structure, migration, and introgression, they are less useful for detecting genes that are actual targets for natural selection [10].

Recently, several methods for detecting sites under selection have been developed. Approaches based on coalescent theory are powerful for detecting loci that have recently undergone natural selection [11]. In coalescent-based methods, DNA polymorphisms are used to detect a particular region that has evolved through selective sweep, balancing selection, or neutral evolution [12]. When attempting to detect selected loci, it is important to consider demographic effects, which can affect entire genomic regions; therefore, the population genetics summary statistics of the target region should be compared with those of many different marker regions within the genome [13]–[15]. Genetic markers such as mitochondrial DNA and microsatellites are not effective genetic reference markers because information obtained from mitochondrial DNA is equivalent to that from a single unrecombined locus, and microsatellites have a more complex mechanism of mutation than that of point mutations. Multiple nuclear markers for non-sequenced species are usually designed using expressed sequence tag (EST) resources, short-insert libraries, cDNA libraries, or multi-genome information from related species [15]. For example, Putta et al. determined EST sequences from two closely-related species of ambystomatid salamander, and used two of these EST sequences to design Polymerase chain reaction (PCR) primers for another ambystomatid salamander species [16]. Creating short-insert libraries can also provide polymorphic markers without genomic information. Using such libraries, Rosenblum et al. obtained 19 markers that have intra-populational polymorphic sites in the eastern fence lizard (*Sceloporus undulatus*) [17], and Lee and Edwards obtained 35 markers for red-backed fairy wren (*Malurus melanocephalus*) to help understand the origin, dispersal, and geographic structure of these species [18]. However, to obtain enough genomic information for population genetic approaches, these methods require many experimental steps.

Comparative genomics approaches do not require many experimental steps, and are effective for the construction of phylogenetic trees based on molecular markers. Some studies have designed universal primers for non-sequenced species by using publically-available genome information [19]–[23] and by taking advantage of regions that are conserved between species. However, because it is more difficult to design primers to amplify polymorphic genomic fragments of less-conserved gene markers, only a few studies have used this method to detect population polymorphisms.

As one of the few studies that designed population genetics markers by comparative genetics, Backstrom et al. provided genome-wide markers in a broad range of avian species by using chicken genome information and zebra finch EST reference sequences [24]. Both intra- and inter-population polymorphic markers were designed, and the group succeeded in designing intraspecific polymorphic markers in intronic regions. Because intronic regions are more polymorphic than exonic regions [25], it is easier to design polymorphic markers in these regions; introns often include insertion/deletion (indel) polymorphisms [25], and if the samples are heterozygous for multiple indel polymorphisms, it is difficult to determine sequences by the Sanger method. Avian genomes have a low microsatellite frequency [26], and the results of Backstrom's study showed that the frequency of indels is lower than that of polymorphic sites, with long indels being especially infrequent [24]. Therefore, it is effective to use intronic markers for population genetics of birds, although the same does not generally apply to other taxa.

In this study, we design primers that amplify DNA fragments flanked by two conserved regions between fully-sequenced species including not only closely related but also distantly related ones (*e.g.,* frog and chicken). The purpose of the present study is to evaluate the effect of the degree of conservation for the sequences in the reference genome on the amplification success and the level of polymorphism.

## Results and Discussion

### Designing PCR primers by using comparative genomics

We studied four target organisms with differing amounts of available genomic information. *Rana ornativentris*, the montane brown frog, has no related species within the same family in which the whole genome has been sequenced. Similarly, the teleost fish *Poecilia reticulata* (guppy) has no related species within the same order, but there are several other species of teleost fish with sequenced genomes. *Anolis sagrei*, the brown anole, is in the same genus as *Anolis carolinensis*, the green anole, for which the complete genome sequence is available. The genome of *Drosophila melanogaster* has been fully sequenced. We designed primers for the non-sequenced and sequenced organisms by comparing genomic sequences of fully-sequenced reference species (see Materials and Methods). We obtained 150 candidate primer pairs for *R. ornativentris*, 700 pairs for *A. sagrei*, 5,168 pairs for *P. reticulata*, and 2,015 pairs for *D. melanogaster*. Therefore, we were able to obtain candidate primer pairs even in situations where a species had only distantly-related reference genomes (Table 1). The primers were located both in exonic and intronic regions for all species studied. The number of primers designed for *P. reticulata* was much larger than that for the other species. One reason for this was that the primers for *P. reticulata* were obtained by comparing the sequences between more than two reference genomes: *i.e.*, the *Oryzias latipes* genome was compared to that of four teleost fishes (*Danio rerio*, *Tetraodon nigroviridis*, *Takifugu rubripes*, and *Gasterosteus aculeatus*). In addition, the primers designed for *P. reticulata* were more frequently intronic than those of other species (Table 1).

**Table 1:** Primer pairs and genome information.

Species	Reference genome species for designing primers		Number of primer pairs	Region of primers			Proportion of primers in exon
	Species A	Species B		Exon^a^	Intron^b^	Both^c^	
*R. ornativentris*	*G. gallus*	*X. tropicalis*	150	57	56	37	0.38
*A. sagrei*	*G. gallus*	*A. carolinensis*	700	289	236	175	0.41
*P. reticulata*	*O. latipes*	*G. aculeatus*	2067	374	451	1242	0.18
		*T. nigroviridis*	1026	172	180	674	0.17
		*T. rubripes*	1993	346	421	1226	0.17
		*D. rerio*	82	29	10	43	0.35
*D. melanogaster*	*D. melanogaster*	*D. ananassae*	205	125	2	78	0.61

a Number of primer pairs located in exonic regions only.

b Number of primer pairs located in intronic regions only.

c Number of primer pairs located in a combination of exonic and intronic regions.

### Features of DNA fragments amplified by the primers

**Figure 1:**
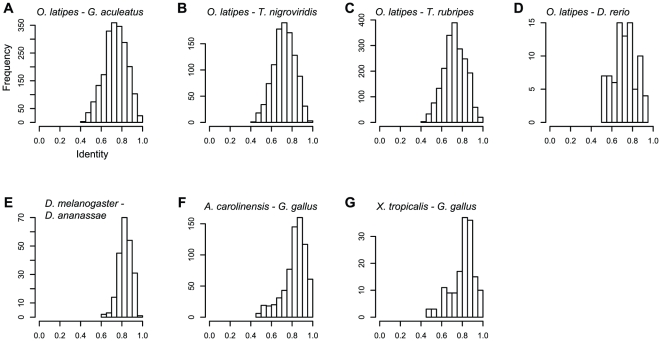
Distribution of sequence identities between orthologous reference genomic regions predicted to be amplified by the primer pairs. The x-axis indicates the identity between orthologous DNA regions predicted to be amplified by primer pairs in the following reference genomes: (A) *Oryzias latipes* and *Gasterosteus aculeatus*; (B) *O. latipes* and *Tetraodon nigroviridis*; (C) *O. latipes* and *Takifugu rubripes*; (D) *O. latipes* and *Danio rerio*; (E) *Drosophila melanogaster* and *Drosophila ananassae*; (F) *Anolis carolinensis* and *Gallus gallus*; and (G) *Xenopus tropicalis* and *G. gallus*.

To estimate the degree of conservation of the DNA fragments predicted to be amplified by our primers, we used the published sequences of the reference genomes to calculate the sequence identities between orthologous DNA regions flanked by primer sites, and compared these to the sequence identities between orthologous genes in the same genomes (Table S1). The primers would be expected to amplify fragments with a wide distribution of identities between reference genomes of fully-sequenced species (Fig. 1). On average, the DNA fragments predicted to be amplified by the primers designed for *R. ornativentris* (based on *G. gallus* and *X. tropicalis* reference genomes), *A. sagrei* (based on *G. gallus* and *A. carolinensis* reference genomes), and *D. melanogaster* (based on *D. melanogaster* and *D. ananassae* reference genomes) (Fig. 1) were more conserved than homologous gene pairs in the corresponding reference genome species (Figure S1), although the identities between the DNA fragments varied considerably. This result could be caused by an enrichment of exonic fragments in these reference genomes (Table 1). Conversely, because the primers for *P. reticulata* (based on *O. latipes* and other teleost reference genomes) were designed to amplify intronic regions, the distribution of fragment identities (Fig. 1) was lower than that of the average gene (Figure S1). Note that, even in the case of primers designed for *P. reticulata*, the identities between exonic fragments were higher than that of the average gene (Figure S2).

By using orthologous genes of reference species and their gene ontology annotation (GOA; see Methods), we examined the functions of genes located within the regions predicted to be amplified by our primers. We found a significant enrichment in some functional categories, particularly for regions predicted to be amplified by primers designed for *P. reticulata* (Table 2). This bias could be caused by our use of distantly-related species (teleost fish taxa, the chicken [*Gallus gallus*] and the Western clawed frog [*Xenopus tropicalis*]) for primer design, because conserved genes have been concentrated as particular identifiable orthologs. Although there was bias in the functional categories represented, the genes were distributed in many functional categories. Thus, it is possible to select gene sets classified in various functions for primer design.

**Table 2:** Functional classes of genes with primers.

GO domains	Species	GO IDs	Term	Obs.	Mean	S.D.	Z score	p-value^a^
biological process	*R. ornativentris*	GO:0006139	nucleobase, nucleoside, nucleotide and nucleic acid metabolic process	7	1.9	1.1	4.6	0.022
	*A. sagrei*	GO:0043170	macromolecule metabolic process	27	12.8	3.0	4.7	<0.001
		GO:0006139	nucleobase, nucleoside, nucleotide and nucleic acid metabolic process	17	6.5	2.4	4.5	0.002
		GO:0007275	multicellular organismal development	1	8.0	2.6	−2.7	0.022
	*P. reticulata*	GO:0050789	regulation of biological process	274	182.8	11.4	8.0	<0.001
		GO:0009058	biosynthetic process	189	119.6	9.5	7.3	<0.001
		GO:0008152	metabolic process	40	83.9	8.2	−5.3	<0.001
		GO:0043170	macromolecule metabolic process	287	217.7	11.8	5.9	<0.001
		GO:0006139	nucleobase, nucleoside, nucleotide and nucleic acid metabolic process	165	112.3	8.8	6.0	<0.001
		GO:0009987	cellular process	386	316.7	13.5	5.1	<0.001
		GO:0007275	multicellular organismal development	63	101.5	9.0	−4.3	<0.001
		GO:0006928	cell motion	2	11.1	3.1	−2.9	0.012
	*D. melanogaster*	GO:0050789	regulation of biological process	15	6.2	2.2	4.0	0.005
molecular function	*R. ornativentris*	GO:0030528	transcription regulator activity	7	0.7	0.7	9.2	<0.001
	*A. sagrei*	GO:0015267	channel activity	5	1.0	0.8	5.1	0.036
	*P. reticulata*	GO:0003676	nucleic acid binding	50	117.5	9.6	−7.0	<0.001
		GO:0030528	transcription regulator activity	66	22.7	4.6	9.3	0.000
		GO:0005515	protein binding	264	185.5	11.5	6.8	<0.001
		GO:0015267	channel activity	43	15.7	3.7	7.4	<0.001
		GO:0015075	ion transmembrane transporter activity	59	31.3	5.3	5.2	<0.001
		GO:0005488	binding	235	292.9	14.0	−4.1	<0.001
		GO:0004872	receptor activity	50	79.0	8.3	−3.5	0.003
		GO:0003824	catalytic activity	7	21.6	4.5	−3.3	0.003
		GO:0016829	lyase activity	17	6.7	2.4	4.3	0.005
		GO:0016740	transferase activity	76	54.9	6.8	3.1	0.047
	*D. melanogaster*	none						
cellular component	*R. ornativentris*	GO:0005634	nucleus	11	3.5	1.6	4.7	<0.001
	*A. sagrei*	GO:0030312	external encapsulating structure	3	0.1	0.4	8.1	0.002
		GO:0005634	nucleus	21	11.3	3.0	3.3	0.013
	*P. reticulata*	none						
	*D. melanogaster*	none						

a The estimated *P* values were adjusted by Bonferroni correction. Only statistically significant functional classes are shown.

### Effect of identity between reference genomic regions on the successful use of primers designed for *R. ornativentris*

**Figure 2:**
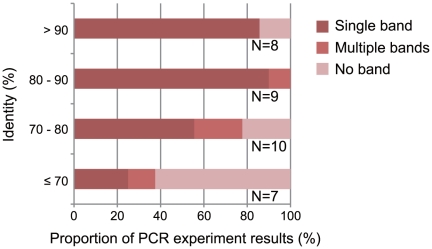
Association between the success-rate of PCR amplification and sequence identities between corresponding reference genomic regions. The x-axis indicates the success rates of PCR amplifications of *Rana ornativentris* sequences using the primer pairs designed from the reference genomes. The y-axis indicates the identity between orthologous reference DNA regions predicted to be amplified by using the primers. “Single band” indicates a successful PCR with a single band amplified; “multiple bands” indicates an unsuccessful PCR with more than two bands or smeary band; and “no band” indicates amplification failure. The number of primer pairs is shown at right of each bar.

**Figure 3:**
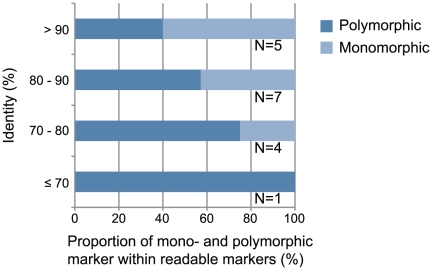
Association between presence of polymorphisms in PCR products and identities between reference genomic regions. The x-axis indicates the proportion of mono- and polymorphic markers amplified by using the primer pairs designed for *Rana ornativentris*. Only readable sequences were included in the analysis. The y-axis indicates the identity between reference genomic regions predicted to be amplified by the primers. The number of primer pairs is shown at right of each bar.

We selected a small subset of the primers for further experiments. First, we used 34 primer pairs designed for *R. ornativentris* to examine the range of sequence identities between the reference genomes of two sequenced species (*G. gallus* and *X. tropicalis*). To avoid chromosomal bias, we selected one or two primer sets from each chromosome of *G. gallus*. We hypothesized that primers associated with highly-conserved sequences having a high identity between the reference genomes would amplify *R. ornativentris* sequences containing few polymorphisms. The corollary to this is that primers associated with highly-divergent sequences having low identity between the reference genomes would amplify *R. ornativentris* sequences that are more polymorphic; however, DNA amplification would be expected to be less successful for such sequences because the primers may not match as well to the reference genome. Therefore, we examined whether there was an association between the success rate of DNA amplification and the identity between the reference sequences. The results, using primers designed for *R. ornativentris*, showed that the success rate of DNA amplification was high when the identity was high, but not when the identity was low (Fig. 2). We also observed that the success rate of DNA sequencing correlated with the identity values (data not shown). In contrast, readable sequences amplified by primers designed for *R. ornativentris* were more polymorphic when the identity was low than when it was high (Fig. 3). Our results indicate that sequence identity between reference genomes has strong effects on the success of detecting new polymorphic markers in the target genome and should be taken into account during primer design.

**Figure 4:**
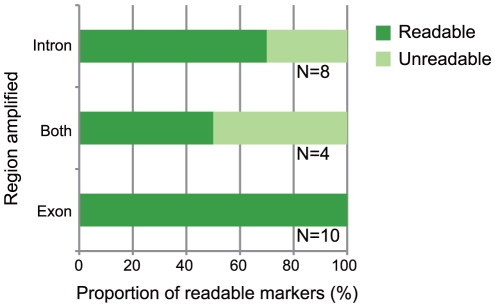
Association between readable sequence and intronic-exonic location of amplified *Rana ornativentris* regions. The x-axis indicates the proportion of *R. ornativentris* markers with readable sequences obtained by using the primer pairs. Only markers that showed a single band after PCR were included in this analysis. “Readable” means that sequencing was successful using PCR primers. “Unreadable” means that sequencing failed and/or insertion/deletion (indel) polymorphisms prevented the sequence from being read. “Intron”, “Both”, and “Exon” indicate the location of the amplified regions: i.e., including only intronic sequences, including both intronic and exonic sequences, and including only exonic sequences, respectively. The number of primer pairs is shown at right of each bar.

Because indel polymorphisms are less frequent in exonic regions than in intronic regions, we expected that primers designed to hybridize to exonic regions would be more likely to amplify readable sequences. By using the primers designed for *R. ornativentris*, we found that a substantial proportion of intronic primer pairs amplified unreadable sequences, whereas all primers designed to amplify exonic regions yielded readable DNA fragments (Fig. 4). Therefore, we primarily selected exonic primers for the other species in our study as described below.

### Amplification of DNA fragments by using primers designed for *A. sagrei*, *P. reticulata* and *D. melanogaster*

On the basis of our results for *R. ornativentris*, we selected 21, 28, and 26 primer pairs for the DNA amplification of *A. sagrei*, *P. reticulata*, and *D. melanogaster* sequences, respectively (sequence identity between reference genomes: 75%–90%). The success rates of DNA amplification using the primer pairs were 65% (22/34 pairs) for *R. ornativentris*, 71% (15/21 pairs) for *A. sagrei*, 58% (27/46 pairs) for *P. reticulata*, and 77% (20/26 pairs) for *D. melanogaster*. The success rate of DNA amplification in *D. melanogaster* was the highest of the four species, most likely because the primers were designed by using the *D. melanogaster* genome itself. The result is consistent with the fact that, in primer design by comparative genomics approaches, the success rate of PCR amplification inversely correlates with divergence time between a target and its reference species (*i.e.*, the smaller the divergence time the higher the success rate of PCR amplification) [15]. Although primers for the other three species had lower success rates than those for *D. melanogaster*, their success rates were sufficiently high to enable the identification of numerous new polymorphic markers (Table S2).

### Sequence analysis of amplified markers

Rates of readable sequences generated by candidate primer pairs were 77% (17/22 pairs) for *R. ornativentris*, 100% (11/11 pairs) for *A. sagrei*, 100% (10/10 pairs) for *P. reticulata*, and 100% (8/8 pairs) for *D. melanogaster*. When the polymorphisms of the sequences were checked, the rates of polymorphic markers within readable sequences were 59% (10/17 pairs) for *R. ornativentris*, 90% (9/10 pairs) for *A. sagrei*, 88% (7/8 pairs) for *P. reticulata*, and 100% (8/8 pairs) for *D. melanogaster* (Table S2). We consider that the rates of readable sequences including polymorphic sites in *D. melanogaster*, *A. sagrei*, and *P. reticulata* were particularly high because the primers for these three species were predominantly designed to hybridize to exonic regions. Our results suggest that exonic primers are more likely to be successful at amplifying readable sequences in all species, regardless of the divergence time between target genome and reference genome.

We obtained many polymorphic markers in exonic regions for all species in this study (Table S2). The exonic regions not only had a higher success rate of readable sequences when compared to intronic regions (Fig. 4), but they also had polymorphic sites in internal regions even though the primer sequences were completely identical to both reference sequences. Analyses based on coalescent theories are very effective for detecting DNA regions that are subject to natural selection and for estimating the demographic history of populations. Our approach is useful for obtaining multi-loci nuclear DNA sequence markers, especially for population genetic studies. In fact, we have conducted coalescent analyses in *P. reticulata* by using the primers to amplify DNA fragments that were then used as neutral nuclear markers (Tezuka A, et al., *unpublished*). We designed primers not only for fully-sequenced species but also for species that have no related sequenced species within the same family or order. Our approach can therefore be applied to all species.

### The advantages of the present method when compared to those using next-generation sequencers

It is possible to detect extensive polymorphic sites by using next-generation sequencers. For example, the RAD-seq approach can obtain more than ten thousand polymorphic markers by using less than half the capacity of one Illumina sequencing run [27]. The markers are effective for use in the detection of natural selection, genome mapping, and population genetics [27], [28]. However, it is not necessarily the case that tens of thousands of polymorphic markers are required for population genetics. The detection of polymorphic markers by RAD-seq involves large costs and a great deal of time compared to our economical approach, particularly for non-sequenced organisms, because the next-generation sequencers read massive numbers of short genomic fragments randomly for Single Nucleotide Polymorphism (SNP) calling.

If RAD-seq and EST sequences for closely-related species are available, primers could be designed based on the sequences that include polymorphic sites. However, our results show that the use of conserved sequences for primer design increases the PCR success rate (Fig. 2), and this advantage would cancel out the benefit of designing primers to polymorphic sites. Our approach using comparative genomics considers the identity between two reference sequences (Figure S1). In addition, our approach has broad utility when distantly-related species are used for the primer design: the primers generated for the guppy could work for other fish; the primers designed for montane brown frog could work for other frogs and birds; and the primers designed for the anole lizard could work for other lizards and birds (Table S2). In contrast, primers designed based on RAD-seq data for a particular organism might be limited to that organism. Our approach provides polymorphic markers that are not dependent on the genetic distance to the reference sequence.

Next-generation sequencers provide a large amount of genomic information. The number of available whole-genome sequences is increasing rapidly; for instance, some comprehensive genome projects such as Genome 10K Project (http://www.genome10k.org) and i5K Genome Project (http://arthropodgenomes.org/wiki/i5K) are in progress. The increased number of genomic sequences will allow us to develop polymorphic markers for species in taxa for which there is currently poor genomic information. Until further technical innovation drastically reduces the costs of next-generation sequencing, our easier and more affordable method will continue to be useful for the detection of polymorphic markers in non-sequenced species.

## Materials and Methods

### Genome and gene sequences of fully-sequenced species

We obtained genome sequences and unspliced gene sequences for fully-sequenced species (*X. tropicalis*, *G. gallus*, *A. carolinensis*, *D. rerio*, *G. aculeatus*, *O. latipes*, *T. nigroviridis*, *T. rubripes*, *D. melanogaster*, *and Drosophila ananassae*) from Ensembl release 56 (http://www.ensembl.org). The locations of exons and introns were downloaded from Ensembl release 56. The unspliced gene sequences of *A. carolinensis* were obtained from Ensembl release 55 because complete information was not available in Ensembl release 56.

### Orthologous gene pairs

One-to-one orthologous gene pairs (*X. tropicalis*–*G. gallus*, *A. carolinensis*–*G. gallus*, *O. latipes*–*G. aculeatus*, *O. latipes*–*T. nigroviridis*, *O. latipes*–*T. rubripes*, *O. latipes*–*D. rerio*, *and D. melanogaster*–*D. ananassae*) were downloaded from Ensembl release 56 (http://www.ensembl.org). Orthologs with a one-to-many or many-to-many relationship were not used in order to minimize the likelihood of choosing genes with lineage-specific small-scale gene duplication; this would prevent the primers for the genes from amplifying more than one genomic fragment during the PCR.

### Primer design using fully-sequenced species

We conducted an all-to-all BLAST search (blastn) for the unspliced gene sequences of one species from each pair used in our study (*X. tropicalis*–*G. gallus*, *A. carolinensis*–*G. gallus*, *O. latipes*–*G. aculeatus*, *O. latipes*–*T. nigroviridis*, *O. latipes*–*T. rubripes*, *O. latipes*–*D. rerio*, *and D. melanogaster*–*D. ananassae*) to identify perfectly identical genomic regions (≥20 bp) and to define conserved regions (CRs). We searched for two CRs within a 300–700-bp interval for each of the species pairs. A dummy sequence (NNNN(AATT)_25_NNNN) was inserted between the two CRs prior to conducting primer3 analysis in EMBOSS [29] to precisely design PCR primers based on the CRs. Once forward and reverse PCR primers were successfully designed, a BLAST search (blastn) for the primer sequences was performed by using the whole genomes of both members of the species pair, and the results were checked to ensure that there were no homologous regions in the genomes, except for the primer sequences themselves (E-value <0.1). We extracted sequences from interval regions (IRs) between the CRs for each of the reference species pairs. Sequences were aligned using ClustalW [30], and the identity between the reference IRs was estimated using the aligned reference sequences after removing any loci with an unknown nucleotide (N) if any (Fig. 1). We classified the sequences into three categories: exonic, intronic, or both. In cases where many PCR primers were generated, 21–34 primer pairs were selected for further study on the basis of the following conditions: (i) The primers spanned the whole genome. Chromosomal bias was eliminated by using the complete genome-sequenced species information to determine the location of the primers. (ii) The primers would be predicted to amplify fragments with a wide range of identity between the reference genomes (75%–90%). (iii) The primers would be predicted to amplify exonic and intronic regions. Structural mutations rarely occur in exons; however, it is more difficult to detect polymorphisms in exons than in introns.

### Gene Ontology

Gene Ontology (GO) IDs and GO “slim” annotations for biological processes, molecular function, and cellular components of *D. melanogaster*, chicken, and zebrafish were downloaded from ftp://ftp.geneontology.org/pub/go/gene-associations/ and ftp://ftp.geneontology.org/pub/go/GO_slims, respectively. We excluded the GO ID GO:0008150 (biological process unknown). We used chicken genes as montane brown frog or anole orthologs, and zebrafish genes as guppy orthologs. The frequency of each GO ID assigned to genes with at least one primer site was counted. We calculated the *P* value for each GO ID by comparing the observed frequency in the dataset with the expected frequency determined using a hypergeometric distribution of all genes with at least one GO ID. The estimated *P* values were adjusted by Bonferroni correction. Significantly under- or over-represented GO IDs for genes containing primer sites are listed in Table 2.

### Samples and DNA extraction

We analyzed 48 eggs sampled from wild breeding sites of the montane brown frog (*R. ornativentris*). We collected 8 egg masses per site, sampled one egg per egg mass, and raised it to the stage before metamorphosis. All larvae were killed by lethal doses of anesthesia (tricaine methanesulphonate, Tokyo Chemical Industry Co., Ltd., Japan) and then preserved in 99.5% ethanol at −20°C. We isolated DNA from the tail of the larvae. Muscle tissue from 10 brown anoles (*A. sagrei*) was sampled from 5 wild populations in Cuba. All samples were fixed in 99.5% ethanol at −20°C. Tissue samples from 80 guppies (*P. reticulata*) collected from 10 wild populations on the island of Trinidad and Tobago were collected and fixed with 90% ethanol at −20°C. Whole body samples were taken from 80 fruit flies (*D. melanogaster*) collected from 8 wild populations in Japan, and fixed with 99.5% ethanol at −20°C. Total DNA isolation of all species was performed according to the cetyltrimethylammonium bromide protocol [31].

### PCR and sequence analysis

PCR analyses were conducted using the candidate primers designed for this study. Amplification reactions were performed in a volume of 50 µL containing 2.5 µL DNA, 5.0 µL 10× ExTaq Buffer, 1 µM dNTP mixture, 0.5 µM MgCl_2_, 1 unit ExTaq (Takara, Shiga, Japan), and 0.5 µM each primer. The amplification conditions were as follows: an initial step of 2 min at 94°C, and then 30 cycles of 30 s at 94°C, 30 s at 60°C, and 1 min at 72°C; and a final step of 15 min at 72°C. The PCR products were purified and cleaned by conducting polyethylene glycol precipitation.

All PCR products were sequenced by using an ABI 3130 Genetic Analyzer (Applied Biosystems, Warrington, UK). The sequencing reactions were carried out by using a BigDye Terminator v3.1 Cycle Sequencing Ready Reaction Kit (Applied Biosystems) under the following conditions: an initial step of 1 min at 96°C, and then 45 cycles of 30 s at 96°C, 5 s at 50°C, and then a final step of 4 min at 72°C. The primers used for the initial amplification were used subsequently for sequencing. Sequencing reaction products were purified and cleaned by performing ethanol precipitation. The sequences obtained were edited and aligned using ClustalX [32] and Se-Al software [33].

### Evaluation of primers

To determine whether the primers would work for PCR and sequencing analysis, we conducted empirical experiments composed of three steps: identification of amplified PCR fragments, selection of readable sequences, and examination of genetic polymorphisms in those sequences. The criterion used for a successful PCR was the obtainment of a single amplification fragment that was close to the expected length (300–700 bp) and detectable in all the samples in the set. The criterion used for selecting readable sequences was that they were readable by using the original PCR primers (at least one-sided primer). For these two steps, we used one individual from each of five different frog populations, one individual from each of five different anole populations, one individual from each of two different guppy populations, and two individuals from wingless strains of *D. melanogaster*. The criterion used to select sequences with genetic polymorphisms was that all sequences obtained using a primer pair should contain at least one polymorphic site.

## Supporting Information

Figure S1 Distribution of identities between orthologous gene pairs in reference genomes. The x-axis indicates the identity between orthologous gene pairs in the following reference genomes: (A) *Oryzias latipes* and *Gasterosteus aculeatus*; (B) *O. latipes* and *Tetraodon nigroviridis*; (C) *O. latipes* and *Takifugu rubripes*; (D) *O. latipes* and *Danio rerio*; (E) *Drosophila melanogaster* and *Drosophila ananassae*; (F) *Anolis carolinensis* and *Gallus gallus*; and (G) *Xenopus tropicalis* and *G. gallus*.
(EPS)

Figure S2 Distribution of identities between exonic regions of reference species. The x-axis indicates the identity between exonic fragments predicted to be amplified from the reference genomes listed below: (A) *Oryzias latipes* and *Gasterosteus aculeatus*; (B) *O. latipes* and *Tetraodon nigroviridis*; (C) *O. latipes* and *Takifugu rubripes*; (D) *O. latipes* and *Danio rerio*; (E) *Drosophila melanogaster* and *Drosophila ananassae*; (F) *Anolis carolinensis* and *Gallus gallus*; and (G) *Xenopus tropicalis* and *G. gallus*.
(EPS)

Table S1 Sequence identities between pairs of DNA fragments and orthologous gene pairs from 2 reference species.
(DOC)

Table S2 Primers for polymorphic markers.
(XLS)
