# Supplementary material for: Comprehensive Primer Design for Analysis of Population Genetics in Non-Sequenced Organisms
Source: PLoS One. 2012 Feb 24;7(2):e32314. doi: 10.1371/journal.pone.0032314 (PMC3289539; doi:10.1371/journal.pone.0032314)
Supplement: Table S1 — Sequence identities between pairs of DNA fragments and orthologous gene pairs from 2 reference species. (DOC) [file pone.0032314.s003.doc]

Table S1. Sequence identities between pairs of DNA fragments and orthologous gene pairs from 2 reference species

| Species | Reference genome species for designing primers | | Identity average | | |
| --- | --- | --- | --- | --- | --- |
|  | Species A | Species B | DNA fragments^a^ | Exon regions^b^ | Whole genes^c^ |
| *R. ornativentris* | *G. gallus* | *X. tropicalis* | 0.81 | 0.82 | 0.69 |
| *A. sagrei* | *G. gallus* | *A. carolinensis* | 0.83 | 0.85 | 0.76 |
| *P. reticulata* | *O. latipes* | *G. aculeatus* | 0.74 | 0.85 | 0.79 |
|  |  | *T. nigroviridis* | 0.73 | 0.85 | 0.77 |
|  |  | *T. rubripes* | 0.73 | 0.82 | 0.77 |
|  |  | *D. rerio* | 0.73 | 0.79 | 0.70 |
| *D. melanogaster* | *D. melanogaster* | *D. ananassae* | 0.84 | 0.85 | 0.77 |

^a^Average identity between the reference genomic regions that were used for designing primers.

^b^Average identity between reference exonic regions that were used for designing primers.

^c^Average identity between all orthologous gene pairs in the reference genomes.
